# Use of Fourier Transform Infrared Spectroscopy for Monitoring the Shelf Life and Safety of Yogurts Supplemented With a *Lactobacillus plantarum* Strain With Probiotic Potential

**DOI:** 10.3389/fmicb.2021.678356

**Published:** 2021-06-28

**Authors:** Olga S. Papadopoulou, Anthoula A. Argyri, Varvara Kounani, Chrysoula C. Tassou, Nikos Chorianopoulos

**Affiliations:** Institute of Technology of Agricultural Products, Hellenic Agricultural Organization — DIMITRA, Athens, Greece

**Keywords:** yogurt, probiotic, *Listeria monocytogenes*, Fourier transform infrared spectroscopy (FTIR-ATR), data analytics, quality, safety

## Abstract

The current study aimed to explore the performance of a probiotic *Lactobacillus* strain as an adjunct culture in yogurt production and to assess Fourier transform infrared spectroscopy as a rapid, noninvasive analytical technique to evaluate the quality and the shelf life of yogurt during storage. In this respect, bovine milk (full-fat) was inoculated with the typical yogurt starter culture without (control case) or with the further addition of *Lactobacillus plantarum* T571 as an adjunct (probiotic case). The milk was also inoculated with a cocktail mixture of three strains of *Listeria monocytogenes* in two different initial levels of inoculum, and the fermentation process was followed. Accordingly, yogurt samples were stored at 4 and 12°C, and microbiological, physicochemical, molecular, and sensory analyses were performed during storage. Results showed that the lactic acid bacteria exceeded 7 log CFU/g during storage in all samples, where the probiotic samples displayed higher acidity, lower pH, and reduced counts of *Lb. monocytogenes* in a shorter period than the control ones at both temperatures. Pulsed-field gel electrophoresis verified the presence of the probiotic strain until the end of storage at both temperatures and in adequate amounts, whereas the survival and the distribution of *Listeria* strains depended on the case. The sensory evaluation showed that the probiotic samples had desirable organoleptic characteristics, similar to the control. Finally, the spectral data collected from the yogurt samples during storage were correlated with microbiological counts and sensory data. Partial least squares and support vector machine regression and classification models were developed to provide quantitative estimations of yogurt microbiological counts and qualitative estimations of their sensory status, respectively, based on Fourier transform infrared fingerprints. The developed models exhibited satisfactory performance, and the acquired results were promising for the rapid estimation of the microbiological counts and sensory status of yogurt.

## Introduction

For almost three decades now, the popularity of the probiotic concept and the awareness about the health benefits of consuming probiotic products continue to increase, and therefore, high demand for fermented dairy products fortified with probiotic microorganisms is still arising (Saad et al., 2013; Casarotti et al., 2014). In this respect, considerable research is being carried out for the production of probiotic yogurts with new probiotic lactobacilli strains isolated from traditional fermented foods, as the isolation and screening of microorganisms from natural sources have been a powerful tool to obtain new strains that are beneficial and genetically stable for industrial use (Solieri et al., 2014). Moreover, scientific pieces of evidence indicated that probiotic lactobacilli inhabiting fermented foods could have beneficial properties for well-being and health maintenance (Morelli, 2007). For instance, Zhou et al. (2019) studied the effect on yogurt fermentation and storage after adding *Lactobacillus helveticus* H9, a probiotic bacterium isolated from fermented yak milk with known antihypertensive activity, and observed that although probiotic yogurt scores were lower in sensory assessment, the activity of the antihypertensive angiotensin-converting enzyme inhibitor was higher compared with control samples, resulting to health benefits to the consumer. Moreover, Rutella et al. (2016) used two probiotic strains (*Lactobacillus casei* PRA205 and *Lactobacillus rhamnosus* PRA331) previously isolated from ripened Parmigiano–Reggiano cheeses to study their survival and the release of antihypertensive and antioxidant peptides during yogurt fermentation and cold storage. The results demonstrated that *Lb. casei* PRA205 could be used as an adjunct culture for producing bifunctional yogurt. Maragkoudakis et al. (2006) examined the performance of two potential probiotic lactobacilli strains (*Lactobacillus plantarum* ACA-DC 146 and *Lactobacillus paracasei* subsp. *tolerans* ACA-DC 4037) in fermentation and storage of traditional Greek set-type yogurt, whereas Saxami et al. (2016) studied two lactobacilli strains (*Lactobacillus pentosus* B281 and *Lb. plantarum* B282, previously isolated from naturally fermented table olives) in mono and mixed cultures, in the production of probiotic yogurt. In both studies, results confirmed that these strains could be promising candidates for inclusion as adjuncts cultures to manufacture yogurt with potential probiotic properties.

Yogurt is inhibitory to foodborne pathogens due to low pH (4.0–4.5), low storage temperature, and the possible production of organic acids and bacteriocins by the fermenting microorganisms, which can act as bactericidal/bacteriostatic (Mufandaedza et al., 2006; Ben Slama et al., 2013). Although *Listeria monocytogenes* is not frequently found in yogurt, it is a ubiquitous pathogen that can survive over a wide range of stress conditions and is recognized as a post-processing contaminant of major concern in dairy products such as yogurt (Tirloni et al., 2015). Indeed, *Lb. monocytogenes* has been reported to be present in food processing environments, especially in the dairy plant environment, where it could enter through contaminated raw milk and spread to food contact surfaces through inadequate hygiene practices, unhygienic design of equipment, inappropriate personnel movements, and contaminated contact materials (Almeida et al., 2013; Muhterem-Uyar et al., 2015). Thus, several studies dealt with the survival of *Lb. monocytogenes* after artificial contamination of yogurt, before or after fermentation, and observed that inactivation during storage depended on many factors (Massa et al., 1991; Akkaya et al., 2009; Tirloni et al., 2015; Al-Nabulsi et al., 2016; Szczawiński et al., 2016).

Fourier transform infrared (FTIR) spectroscopy is an analytical technique that can obtain spectra from a wide range of different compounds. FTIR can be used as a quantitative control method in the food industry, as it is a rapid and noninvasive analytical technique that does not require long and laborious sample preparation and special consumables or skilled labor and, in conjunction with attenuated total reflectance (ATR) technology, allows users to collect full spectra in a few seconds from solids, liquids, semisolids, and thin films (Ammor et al., 2009; Rodriguez-Saona and Allendorf, 2011). Over the last decade, significant research has been carried out using FTIR in conjunction with multivariate analysis techniques (principal component analysis, discriminant function analysis, partial least squares (PLS) regression, artificial neural networks, and/or support vector machines) for the detection, identification, and quantification of spoilage in a variety of foods products. For instance, FTIR coupled with multivariate data analysis was used to monitor microbial spoilage of different types of meat (Papadopoulou et al., 2011; Argyri et al., 2013; Panagou et al., 2014; Fengou et al., 2019a) and microbiological quality of fish products (Saraiva et al., 2017; Fengou et al., 2019b). On the other hand, there is limited scientific literature available for the evaluation of the shelf life of dairy products using FTIR, as most of the articles found are dealing with the feasibility of FTIR to determine chemical composition, adulteration, and authenticity of dairy products (De Marchi et al., 2018). Nevertheless, FTIR spectral data were used in tandem with PLS analysis to estimate the microbiological counts and sensory status during storage of Feta cheese (Papadopoulou et al., 2018). With regard to yogurt, different data analytical techniques were applied to correlate FTIR spectral data for the detection of milk fat adulteration (Temizkan et al., 2020), or protein adulteration in yogurts (Xu et al., 2013), to determine sugar content in commercial yogurts and yogurt drinks (Khurana et al., 2008) or protein content in a variety of commercial types of yogurt (Khanmohammadi et al., 2009) and finally, to estimate the nutritional parameters of commercial yogurts (Moros et al., 2006).

Based on the above, the current study aimed to evaluate the performance of *Lb. plantarum* T571 strain with probiotic potential, as adjunct culture in yogurt production and storage, and its effect on safety regarding the survival of *Lb. monocytogenes*. Hence, cow’s milk was inoculated with the typical yogurt culture (control case) and with the further addition of the probiotic strain *Lb. plantarum* T571 (probiotic case), and samples of both cases were also inoculated with an *Lb. monocytogenes* cocktail culture (three strains) using two different initial inoculum levels (3 and 7 log CFU/ml). All the products were stored at 4 and 12°C, for a total storage period of 30 and 21 days, respectively. Microbial, physicochemical, and sensory analyses (samples without *Listeria* inoculations) were performed during storage for all cases. Additionally, strain distribution, i.e., LAB and *Lb. monocytogenes*, was evaluated during storage using molecular tools. Finally, the feasibility of FTIR-ATR in tandem with data analytics was investigated to evaluate the quality and the shelf life of yogurt during storage in an attempt to correlate microbiological counts and sensory scores with the spectral data.

## Materials and Methods

### Microbial Cultures

Yogurt was manufactured using the typical yogurt culture (control samples) of *Lactobacillus bulgaricus* (ACA-DC 84) and *Streptococcus thermophilus* (ACA-DC 6) (kindly provided by Laboratory of Dairy Research, Department of Food Science and Human Nutrition, Agricultural University of Athens). Furthermore, for the production of the probiotic yogurt (probiotic samples), the strain of *Lb. plantarum* T571 (culture collection of the Institute of Technology of Agricultural Products, Hellenic Agricultural Organization—DIMITRA), which its probiotic potential was previously explored *in vitro* (Pavli et al., 2016), was mixed with the typical yogurt culture, as it is described later. All the LAB strains were revived from a stock culture stored at −80°C, into 10 ml on MRS broth (MRS broth, 4017292, Biolife, Milano, Italy) for lactobacilli and into 10-ml M17 broth (M17 broth, BK012HA, Biokar Diagnostics, Allonne, France) for *S. thermophilus* and were incubated overnight at 37°C. A subculture was prepared in fresh 10-ml broths of MRS and M17 and incubated at 37°C for 24 h. For the milk inoculation, the cells were harvested by centrifugation (6,000 × *g*, 5 min, 4°C), washed twice with one-fourth strength Ringer’s solution (Ringer solution Tablets, 96724- 100TAB, Merck, Darmstadt, Germany), and resuspended in the same solution to give a final population approximately 6 log CFU/ml in the milk. To confirm the inoculum size, the same dilutions were poured and plated on MRS ISO Agar (MRS Agar, NCM0190A, Neogen, Lansing, MI, United States) and M17 Agar (M17 Agar, 4017192, Biolife) for lactobacilli and *S*. *thermophilus*, respectively.

A cocktail of three strains of *Lb. monocytogenes* was used to inoculate the milk at two different initial inoculum levels (3 and 7 log CFU/ml), in tandem with the LAB strains for both control and probiotic cases. The three strains [Food Microbiology Culture Collection (FMCC)-B129, FMCC-B131, and FMCC-B133] used in the current study have been previously isolated from Greek food industries and were kindly provided by the Laboratory of Microbiology and Biotechnology of Foods, Agricultural University of Athens. A 10-ml Brain Heart Infusion (BHI) broth (LAB049, LabM, Lancashire, United Kingdom) was used to revive the strains from a stock culture stored at −80°C and was incubated subsequently for 18 h at 37°C. A subculture from each strain was prepared in a fresh 10-ml BHI broth and incubated following the same conditions. The monocultures of the aforenoted strains were centrifuged (6,000 × *g*, 5 min, 4°C), washed twice with one-fourth strength Ringer’s solution, and resuspended in 10-ml Ringer solution. The strains were inoculated in the milk as a cocktail so, to prepare the three strains cocktail, each monoculture was mixed in equal volume, and this final solution containing the three strains was used to inoculate the milk at the two different inoculum levels (3 and 7 log CFU/ml) for both control and probiotic case. To confirm the inoculum size, the appropriate dilutions were spread plated on Palcam Agar (Palcam Agar, BK145HA, Biokar Diagnostics, Allonne, France).

### Yogurt Production

The yogurt samples were produced at the Institute of Technology of Agricultural Products, Hellenic Agricultural Organization—DIMITRA (Lycovrissi, Greece). Full fat (3.5%) pasteurized and homogenized bovine milk with initial pH of approximately 6.7 purchased from a local market (Athens, Greece) was inoculated with the typical yogurt starter culture (*Lb. bulgaricus* and *S. thermophilus*) (control case) and with further addition of the probiotic culture *Lb. plantarum* T571 as adjunct culture (probiotic case), using equal inoculum sizes of each strain of approximately 6 log CFU/ml population levels. The inoculated milk was placed in 100-ml sterile screw cap vessels, and the samples were consequently incubated under controlled isothermal conditions at 42°C in high precision (±0.5°C) incubators (Friocell, MMM Medcenter Einrichtungen GmbH, Munich, Germany) until a firm coagulum was formed and the pH value of the yogurt reached 4.6. Milk without (control samples) or with the addition of *Lb. plantarum* T571 strain (probiotic samples) was also inoculated with the three strains cocktail of *Lb. monocytogenes* at two initial inoculum levels (3 and 7 log CFU/ml), and the same fermentation process was followed, as described earlier. After fermentation (42°C, 5 h, pH 4.6), the samples were stored at 4 and 12°C, until the end of shelf life. For safety reasons, inoculation of the milk (control and probiotic cases) with the three *Listeria* strains took place in a separate laboratory inside a laminar flow to avoid contamination with the non-inoculated samples. Consequently, the inoculated milk was incubated in separate incubator chambers and then stored in different chambers from the non-inoculated samples (pathogen-free samples) until the end of the experiment. The samples stored at 4°C were analyzed at 0 (after the end of the fermentation process), 2nd, 5th, 9th, 12th, 16th, 19th, 23rd, 26th, and 30th day, whereas samples stored at 12°C were analyzed at 0 (after the end of the fermentation process), first, 4th, 7th, 10th, 13th, 14th, 17th, 19th, and 21st day. All experiments were carried out using two different milk batches with three replicates each (2 × 3).

### Microbial Enumeration

The microbial analyses of yogurt samples were carried out until the end of storage at 4 and 12°C. Representative 25-g portions of triplicate yogurt samples (from each batch) were aseptically added to 225 ml of sterilized one-fourth Ringer’s solution and subjected to serial dilutions in the same diluent. Consequently, 0.1- or 1-ml samples were spread or poured on the following agar media: Tryptic Glucose Yeast Agar (TGY Agar, 4021452, Biolife) for the enumeration of total viable counts (TVCs), incubated at 30°C for 48–72 h; MRS ISO Agar (Neogen) overlaid with the same medium for the enumeration of LAB, incubated at 37°C for 48–72 h; M17 Agar (Biolife) overlaid with the same medium for the enumeration of lactococci and *S. thermophilus*, incubated at 37°C for 48 h; Rose Bengal Chloramphenicol Agar (RBC Agar, BK151HA, Biokar Diagnostics) for yeasts and molds, incubated at 25°C for 72 h; and Palcam Agar (Biokar Diagnostics) with Palcam selective supplement (BS00408, Biokar Diagnostics) for the enumeration of *Lb. monocytogenes*, incubated at 37°C for 24 and 48 h (to lower the detection limit of the pathogen to 1 log CFU/g, 1 ml of the samples’ first decimal dilution was spread plated to three Petri dishes). Additionally, to ensure the presence/absence of *Lb. monocytogenes* in the samples, the enrichment method was followed according to ISO 11290-1:1996/Amnd1:2004. In more detail, for the yogurt samples with the low (3 log CFU/mL) initial inoculum level, enrichment was applied throughout storage, whereas for the high inoculum (7 log CFU/mL), enrichment was applied when the pathogen was found below 3 log CFU/g. Overall, 720 samples were analyzed (2 batches/3 replicates/10 sampling points/6 cases/2 temperatures).

### Isolation of Lactic Acid Bacteria and *Listeria* Cells

Approximately 20% of the colonies from the highest countable dilution of MRS Agar (LAB isolates) and from the appropriate countable dilution of Palcam Agar (*Listeria* isolates) were randomly collected. Isolates of *Lb. monocytogenes* were also collected from Palcam Petri dishes (3–10 isolates) from the enrichment procedure when counts were below the detection limit for the enumeration method. Pure cultures included in this study were stored at −80°C in MRS or BHI broth supplemented with 20% (v/v) glycerol (Penta, Radimova, Praha, Czechia) for LAB and *Listeria*, respectively. A total of 120 LAB isolates were retrieved from 4°C (69 isolates) and 12°C (51 isolates) from the highest countable dilution (≥6 log CFU/ml) of MRS Agar plates, and 433 *Listeria* isolates were retrieved from Palcam Agar plates at 4°C (180 isolates) and 12°C (153 isolates). All the isolates were recovered from plates that corresponded to the beginning (day 0, after the end of the fermentation process), middle, and final storage time. Before further analysis, each isolate was grown twice in MRS broth for LAB and BHI broth for *Listeria* strains at 37°C for 24 h, whereas the purity of the cultures was always checked before use on the appropriate agar plates. All isolates were subsequently screened with pulsed-field gel electrophoresis (PFGE).

### Pulsed-Field Gel Electrophoresis

PGFE was applied for typing the *Lb. plantarum* T571 strain and *Lb. monocytogenes* strains, according to Doulgeraki et al. (2010) and Kostaki et al. (2012), respectively. All isolates were digested with the restriction enzyme *Apa*I (10U) (New England Biolabs, Ipswich, MA, United States) according to the manufacturer’s recommendations for 16 h. Restriction fragments were separated in 1% PFGE grade agarose gel (Bio-Rad, Hercules, CA, United States) in 0.5-mM Tris–borate buffer on CHEF-DRIII (Bio-Rad, Hercules, CA, United States) equipment with running parameters as described at Papadopoulou et al. (2018). The obtained restriction profiles were compared with the PFGE fingerprints of the inoculated *Lb. monocytogenes* strains and *Lb*. *plantarum* T571 strain.

### Physicochemical Analysis

Titrable acidity (TA) and pH were measured during manufacturing and storage of the yogurt samples after the end of the microbiological analysis. Titratable acidity was measured by titrating 9 g of sample with 0.1-N sodium hydroxide solution using phenolphthalein as the indicator, according to IDF 81: 1997 method, and the results were expressed as % lactic acid. The pH was recorded with a digital pH meter (HI2211, pH/ORPMeter, HANNA instruments, RI, United States) by immersing the glass electrode in the yogurt sample after the end of microbiological analysis.

### Sensory Evaluation

The sensory evaluation in this study was performed by seven people (staff from the laboratory), who were previously trained and tested in evaluating dairy products (Papadopoulou et al., 2018). The sensory assessment was carried out at the same time intervals as for the microbial analyses (samples without *Lb. monocytogenes*). The sensory assessment of the non-inoculated yogurts took place under artificial light in individual booths in a special sensory analysis room allocated in the Institute of Technology of Agricultural Products. The descriptors selected were based on the perception of color, smell, taste, and texture. A three-class evaluation scheme was used in this experiment, in which the first class (0–1, fresh) corresponded to the absence of off-flavors; the second class (1.5–2, marginal) corresponded to the presence of slight acidic taste and smell but with still acceptable quality; and the third class (2.5–3, unacceptable) corresponded to strong acidic taste and odor (unacceptable quality). Overall, 148 yogurt samples were scored by the sensory panel and were discriminated into the three predefined groups as fresh (52), marginal (40), and unacceptable (56).

### Fourier-Transform Infrared Spectroscopy

Yogurt samples were analyzed using a PerkinElmer Frontier FTIR Spectrometer equipped with a deuterated L-alanine doped triglycine sulfate detector with a KBr window. FTIR spectra were collected using an AMTIR 45° ATR through plate crystal with a HATR sampling accessory, as described in detail at Papadopoulou et al. (2018). In brief, the yogurt samples were placed on the AMTIR ATR crystal, and the measurements were collected directly from the sample surface at 25°C, whereas the scans per measurement were set at 10 with a resolution of 4 cm^–1^. The PerkinElmer Spectrum v10.4.2 software controlled the spectrometer to collect spectra over the wavenumber range 4,000 to 870 cm^–1^. Reference spectra were acquired i) by collecting an air background (AB) spectrum from the cleaned blank crystal before the presentation of each sample and ii) by collecting a water background spectrum (WB) from the crystal surface filled with distilled water. At the end of each sampling, the crystal surface was first cleaned with detergent, washed with distilled water, cleaned with ethanol, and finally dried with lint-free tissue at the end of each sampling interval.

Two FTIR spectra replicates were collected from two biological sample replicates (two batches of yogurt) of each trial. A total of 296 FTIR spectra were collected for each measurement type (AB and WB). The range of FTIR spectra that was used for further analysis was between 1,800 and 900 cm^–1^.

### Modeling of the Spectral Data

Data exploration and interpretation were based on multivariate analytical techniques included in the Unscrambler software (version 9.7, CAMO, Norway) and Statistica ver. 8.0 (Statsoft Inc., Tulsa, OK, United States), such as PLS analysis and support vector machines (SVM). Before data analysis, the FTIR spectral data collected between 1,800 and 900 cm^–1^ were background corrected using the standard normal variate transformation. Subsequently, the data were divided into two datasets, one for the training of the model (first batch, *N* = 148) and one for the validation (second batch, *N* = 148).

For the SVM classification, analysis of the normalized spectral data was used as an input in the SVM classification model, and the output was the predefined sensory class (fresh, marginal, and unacceptable), regardless of storage temperature. The radial basis function kernel was used in the SVM classification model to differentiate the sensory quality of yogurt samples regardless of storage temperature, whereas the parameter C (capacity parameter) was optimized using the grid search technique based on k-fold (*k* = 10) cross-validation, as described in detail at Papadopoulou et al. (2013).

For the regression analysis, PLS regression (PLS-R) models and SVM regression (SVM-R) models were built in an attempt to correlate the population of the different microbial groups (TVC, lactic acid bacteria, lactococci, *S*. *thermophilus*, and *Lb. monocytogenes*) or the sensory scores (0–1, 1.5–2, and 2.5–3) with the normalized spectral data.

For the PLS-R models, the dataset was divided into the training and validation dataset, and the leave-one-out cross-validation procedure was applied on the training set only, as described in detail at Papadopoulou et al. (2011, 2018). When using spectral data, it is a usual procedure to select wavenumbers using the regression coefficients (b-coefficients) and provide the most important wavenumbers to refine the PLS-R model (Papadopoulou et al., 2011), an approach that was used in the current study too. For the case of SVM-R, the normalized FTIR data were used as input variables, and the output was the counts (log CFU/g) of each individual microbial group and the sensory scores of the yogurt samples. In that case, the radial basis function kernel was selected for the development of the SVM-R models, and the parameters *C* and ε (insensitive loss function) were optimized based on the grid search technique based on k-fold (*k* = 10) cross-validation (Papadopoulou et al., 2013).

### Performance Indices of the Spectral Data

The criteria for evaluating and comparing the performance of the two regression models (PLS-R and SVM-R) were the root mean square error (RMSE), the coefficient of determination (R^2^), for the actual values *versus* the prediction of the microbiological data, and the sensory scores. In addition, the bias (B*f*) and accuracy (A*f*) factors and the accuracy of prediction (the percentage of samples correctly predicted, i.e., the difference between predicted and observed value is < 1 log CFU/g, of the total number of the samples within the dataset) were used for the microbial counts (Papadopoulou et al., 2011; Mohareb et al., 2016).

To further evaluate the results of the regression (PLS-R and SVM-R) analysis applied for the sensory scores prediction (1, 1.5, 2, 2.5, and 3), the procedure described in Argyri et al. (2013) was followed. The predicted scores were rounded to the closest class membership using a 0.25 cutoff value for predicted values with respect to the closest sensory value. Consequently, a confusion matrix was built for each case (PLS-R and SVM-R), and the performance of the models was assessed by calculating sensitivity and accuracy of prediction. Sensitivity was calculated by dividing the number of correctly classified samples of a specific sensory group by the total number of samples belonging to this specific group. The accuracy of prediction was defined by dividing the number of correctly classified samples into the three predefined sensory groups by the total number of samples analyzed.

### Statistical Analysis

Physicochemical (pH and TA) and microbiological results were analyzed for statistical significance (*P* < 0.05) with t-test and analysis of variance. All experiments were carried out using two different milk batches with three replicates each (2 × 3).

## Results and Discussion

### Microbial Population and Survival of *Lb. plantarum* T571

**FIGURE 1:**
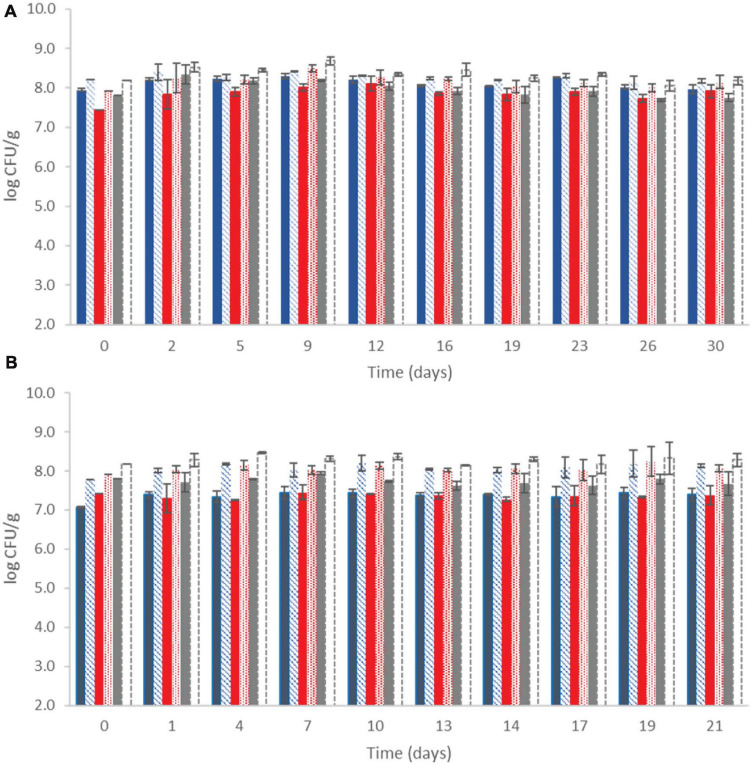
Population of total viable counts (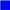), LAB (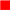), and lactic cocci/streptococci (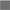) at control samples and Population of total viable counts (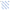), LAB (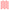), and lactic cocci/streptococci (□) at probiotic samples, during storage of yogurt at 4°C **(A)** and at 12°C **(B)**. Bars represent mean values ± standard deviations. No statistical differences (*P* > 0.05) were observed for all cases except from LAB between probiotic and control samples (*P* < 0.05) at both temperatures during storage.

The microbial population of yogurt samples during storage at both temperatures is shown in Figure 1. It was observed that after the fermentation process, lactococci/*S. thermophilus* (total counts on M17) were found to be above 7.80 ± 0.24 log CFU/g at control samples, whereas the probiotic samples displayed the highest counts (8.16 ± 0.06 log CFU/g). LAB were found at slightly lower population levels, approximately 7.43 ± 0.37 and 8.1 ± 0.20 log CFU/g at the control and probiotic samples, respectively, after the fermentation process (Figure 1). During storage, counts of LAB slightly increased at the probiotic case compared with the control case at both temperatures (*P* < 0.05), reaching population levels of approximately 8.14 ± 0.18 and 8.07 ± 0.10 log CFU/g at 4 and 12°C by the end of storage, respectively. In the control case, after 30 and 21 days of storage at 4 and 12°C, respectively, the population of LAB was found to be lower (7.80 ± 0.04 and 7.37 ± 0.12 log CFU/g, respectively). TVC represented the dominant microbiota in each case, which was LAB and/or lactococci/*S. thermophilus*, depending on the different storage sampling days. No yeasts/molds were detected after enumeration in the current study, and it should also be noted that all the non-inoculated yogurt samples remained *Listeria*-free throughout storage at all cases, as no cells were detected after applying the enrichment method.

According to PFGE analysis, it was demonstrated that up to 100% of the recovered isolates displayed a PFGE profile corresponding to probiotic *Lb. plantarum* T571, at all storage time intervals (day 0, middle, and final storage time) and at both storage temperatures. For a product to be considered probiotic and provide health effects, the population of the probiotic strain must exceed 6 log CFU/g or ml at the time of consumption (Ranadheera et al., 2017). In addition, a recommendation that was recently introduced by Raeisi et al. (2013) suggested that the added probiotic strains must be able to be accurately identified at species or strain level.

In particular, the first criterion was fulfilled, as at probiotic yogurts, the LAB population was maintained over 6 log CFU/g throughout storage, which is consistent with other literature findings (Vinderola et al., 2000; Raeisi et al., 2013). In further detail, Li et al. (2017) studied three *Lb. plantarum* strains on yogurt production and noted that the *Lb. plantarum* viable counts remained stable, exceeding 6.5 log CFU/g along the 28th day in the storage period. The same results were observed for a Greek set-type yogurt produced with the addition of *Lb. paracasei* subsp. *tolerans* ACA-DC 4037, where a population of the probiotic strain was maintained over 7 log CFU/g after 14 days of cold storage (Maragkoudakis et al., 2006). Likewise, Mani-López et al. (2014) observed that the examined probiotic bacteria displayed variability in the viability in the yogurts, maintaining counts ≥ 7 log CFU/ml for different storage periods, which depended on the examined strains. On the other hand, Rutella et al. (2016), who studied two probiotic strains as adjunct cultures in yogurt production and during refrigerated storage, detected that although *Lb. rhamnosus* PRA 331 had higher viable counts after fermentation (approximately 9 log CFU/g), during cold storage, its viable population decreased approximately 2 log CFU/g, whereas the population of *Lb. casei* PRA 205 increased during storage and exhibited the highest population (>8 log CFU/g). Differences in the viability of probiotic strains were also noticed by Vinderola et al. (2000) after the fermentation of yogurt with *Bifidobacterium bifidum* BBI and *Lactobacillus acidophilus* LAI as adjunct cultures. The survival ability of LAI was lower compared with BBI, and also, the selected starter cultures resulted in higher or lower viability of the probiotic strains, indicating the importance of selecting a suitable culture combination for the production of probiotic yogurt (Vinderola et al., 2000).

Regarding the second criterion for the viability of the probiotic strain throughout storage, it was shown that *Lb. plantarum* T571 was viable and in adequate population to confer health benefits. In addition, the results of the current study are consistent with those previously reported from other dairy probiotic products, where the high survival rate of the probiotic strains was evident, with respect to the strain identification with molecular tools (Raeisi et al., 2013; Saxami et al., 2016; Angelopoulou et al., 2017; Sidira et al., 2017; Papadopoulou et al., 2018, 2019).

### Population Dynamics of *Listeria monocytogenes* and Strain Distribution

During fermentation (≈5 h, 42°C) of milk that was co-inoculated with 3 or 7 log CFU/ml initial counts of the three-strain cocktail of the pathogen, the population of the pathogen increased approximately 0.5–1.5 log CFU/g depending on the case. More specifically, regarding the samples inoculated with the low initial inoculum level (3 log CFU/ml), the pathogen population increased to 3.81 ± 0.20 log CFU/g for probiotic samples and 4.35 ± 0.17 log CFU/g for control samples at the end of fermentation. In addition, the samples that were inoculated with the high inoculum (7 log CFU/ml), the corresponding population reached 7.50 ± 0.31 log CFU/g for probiotic samples and 7.90 ± 0.28 log CFU/g for control samples at the end of fermentation (data not presented).

**FIGURE 2:**
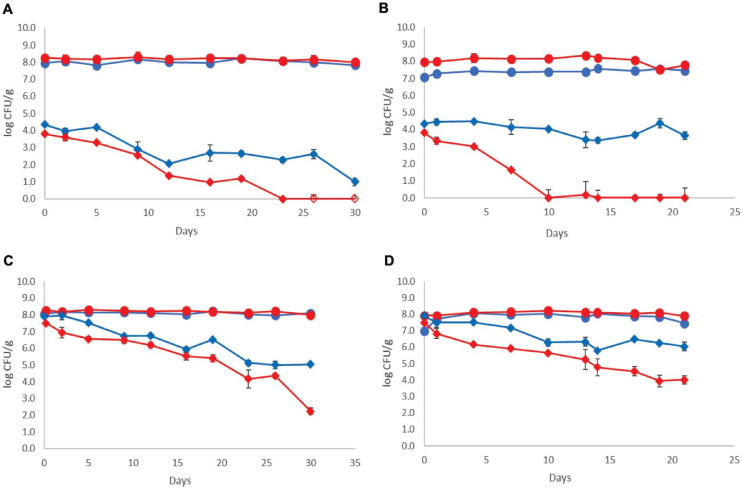
Growth curves of total viable counts without (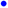) and with (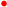) addition of *Lb. plantarum* T571 and growth curves of *Listeria monocytogenes* cocktail strains without (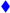) and with (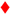) addition of *Lb. plantarum* T571 during storage of yogurt; **(A)** low inoculum, 4°C, **(B)** low inoculum, 12°C, **(C)** high inoculum, 4°C, and **(D)** high inoculum, 12°C. Open symbols (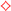) indicate absence of *Lb. monocytogenes* after applying enrichment method. Bars represent mean values ± standard deviations (two biological samples, each sample analyzed three times).

The population dynamics of the pathogen (three-strain cocktail) inoculated at two different initial levels in both control and probiotic cases and stored at 4 and 12°C is presented in Figure 2. The *Listeria* initial counts at the beginning of storage (day 0, after the end of fermentation) were 3.81 ± 0.20 log CFU/g for probiotic samples and 4.35 ± 0.17 log CFU/g for control samples at the low inoculum. During storage at 4°C, the pathogen population decreased below the detection limit of the enumeration method on the 23rd day at the probiotic case and by the end of storage (day 30) at the control case (<1 log CFU/g). Additionally, the probiotic samples were found *Listeria*-free after 26 days, whereas the control samples remained *Listeria*-positive after applying the enrichment method throughout storage at 4°C (Figure 2). During storage of probiotic samples at 12°C, at the low inoculum case, the *Listeria* population decreased to under the detection limit of the enumeration method (<1 log CFU/g) from the 10th day until the end (day 21) of storage (except the 13th day, where in one of the six replicates, the *Listeria* population was 1.10 log CFU/g). On the contrary, the population of the pathogen fluctuated between 3.40 and 4.40 log CFU/g at the control samples until the end of storage at 12°C.

The pathogen’s initial counts at the beginning of storage (day 0, after the end of fermentation) were 7.50 ± 0.31 log CFU/g for probiotic samples and 7.90 ± 0.28 log CFU/g for control samples, at the high inoculum case. During cold storage, the pathogen’s population decreased at both probiotic and control samples. More specifically, an approximately 5 log reduction for the probiotic case was observed by the end of cold storage (day 30), whereas in the control case, the reduction of the pathogen population was lower (approximately 3 log). However, at all samples, the pathogen population remained above the enumeration detection limit. Consequently, at 12°C, a reduction of the pathogen population throughout storage was also observed, which was higher at the probiotic samples (3.5 log reduction) and lower at the control samples (2 log reduction). The pathogens’ population constantly decreased during storage at 12°C from 7.50 ± 0.31 to 4.03 ± 0.36 log CFU/g at probiotic samples. At control samples, the population of the pathogen fluctuated between 6.28 and 5.79 log CFU/g until the end of storage at 12°C.

Regarding the population of the rest of the examined microbiota, the population levels were comparable with the samples produced without the pathogen, at both the control and probiotic yogurt samples and both temperatures. Thus, the dominant microbiota was LAB and/or lactococci/*S. thermophilus* (depending on the different storage sampling days), where their population was estimated at over 7.50 log CFU/mL during the whole storage period of the yogurt samples at 4 and 12°C. In addition, yeasts/molds were not detected in the samples in all cases.

**FIGURE 3:**
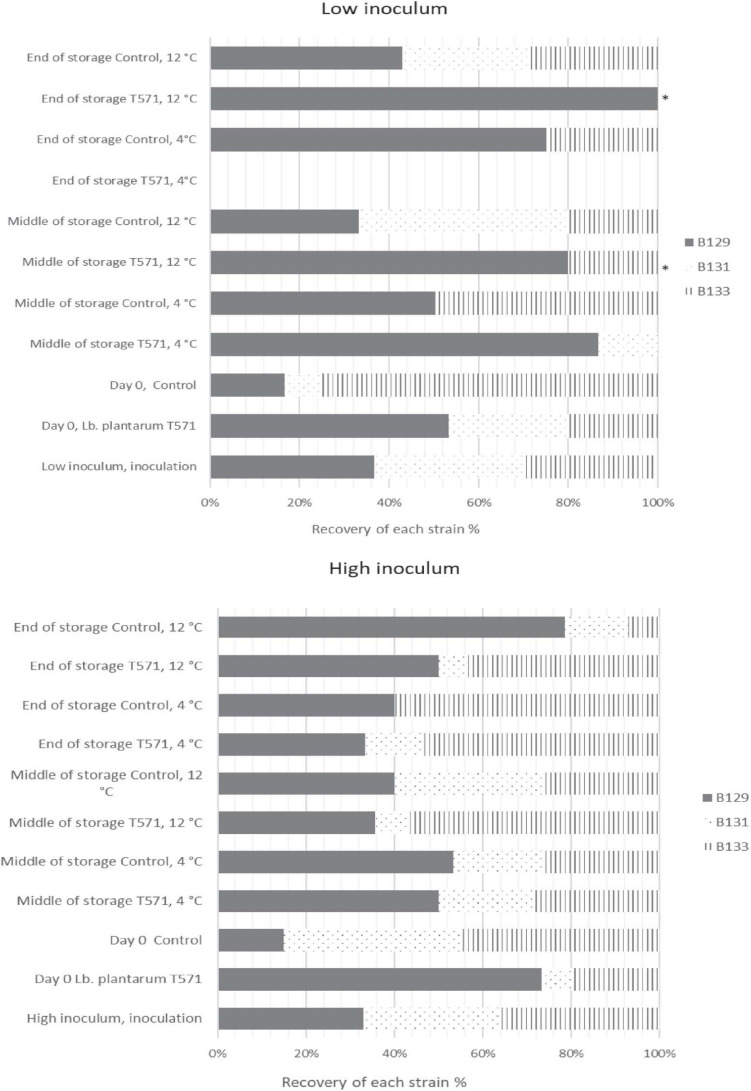
Distribution of *Listeria monocytogenes* isolates (FMCC-B129: dark shade, FMCC-B131: dots, and FMCC-B133: vertical lines) recovered during storage of yogurt samples based on PFGE profiles for low and high inoculum. ^∗^Pathogen detected after applying enrichment method.

The distribution of the 433 isolates retrieved from Palcam Petri dishes based on PFGE profiles is presented in Figure 3. In more detail, the milk was initially inoculated with an approximately 1:1:1 ratio of the strains (FMCC-B129, FMCC-B131, and FMCC-B133) at each case, as shown in Figure 3. After the fermentation process (day 0) at the low inoculum, strain B131 showed the lowest contribution in both the control (8.33%) and probiotic (26.67%) cases. The strain B133 was recovered at the highest percentage at control samples (75%), whereas the B129 recovery was the highest at probiotic samples (53.33%). At the middle storage point at 4°C, the strains B129 (86.67%) and B131 (13.33%) were recovered from probiotic samples, whereas the B129 and B133 strains shared the same recovery percentage (50%) at control samples. By the end of storage, the probiotic yogurt was pathogen-free, whereas, for control samples, strain B129 displayed the highest recovery percentage (75%), followed by B133 (25%). At the middle and final storage point of probiotic samples at 12°C, the pathogen was detected only in samples analyzed by the enrichment method, with the B129 strain being observed at both points and the B133 being observed only at the middle storage point. However, it should be underlined that for the isolates that were recovered after applying the enrichment method, the percent presence of the strains is random, and the appearance of the strains in the figures should be interpreted only as qualitative information instead of percent contribution in the samples. The latter can be enhanced by previous studies that revealed that the enrichment ISO method could be biased on the selectivity of *Listeria* strains (Zilelidou et al., 2016). Finally, at control samples, at the middle and at the final storage point, the distribution of the strains was B129 (approximately 33%-middle- and 42% -final-) > B131 (approximately 46 and 28%) > B133 (approximately 20 and 28%), respectively.

Subsequently, at the high inoculum level and after fermentation, the strain B131 exhibited the lowest recovery percentage (6%) at the probiotic case, whereas B129 presented the lowest recovery rate (15%) at the control case. At the middle storage point at 4°C, all of the three inoculated strains were recovered for both the probiotic and control cases, whereas at the final storage point, the strain B131 was not detected at control samples (Figure 3). However, at 12°C, B129 and B133 were the strains mostly recovered from the middle and final storage points of all cases.

Up to now, several studies have been carried out monitoring the survival of inoculated *Lb. monocytogenes* during the fermentation and storage of various dairy products to examine the potential impact on food safety. At former works, the population of the pathogen increased after the fermentation process, providing insights into the risk of pre-fermentation contamination (Gulmez and Guven, 2003; Al-Nabulsi et al., 2016; Papadopoulou et al., 2018, 2019). For instance, Gulmez and Guven (2003) investigated the behavior of *Lb. monocytogenes*, *Escherichia coli* O157:H7, and *Yersinia enterocolitica* O3 during yogurt fermentation and storage, and the results showed that all the examined pathogens increased during fermentation approximately 1 log CFU/ml, whereas *Lb. monocytogenes* and *E. coli* O157:H7 survived throughout the shelf life. On the other hand, Massa et al. (1991) inoculated cow’s milk with two inoculation levels of *Lb. monocytogenes* and observed an approximately 1 log reduction in viable *Listeria* cells when pH reached 4.5 and a further reduction during shelf life. Concurrently, another study examined the behavior of *Lb. monocytogenes* (3 and 6 log CFU/ml initial inoculum) during milk fermentation with the addition of a bacteriocin-producing *S. thermophilus* B and observed that *Listeria* was below the detection limit within 24 h of processing, whereas at control samples (culture without bacteriocin), *Listeria* reduction was 1 log after 24 h (Benkerroum et al., 2002). In another study dealing with *Listeria* artificial post-contamination on unflavored and flavored strawberry yogurts, researchers observed a reduction in *Listeria* viable counts during shelf life, although *Listeria* was found very resistant and was always detected until the end of the shelf life of the products (Tirloni et al., 2015). Regarding the survival rate of *Listeria* spp. on different temperatures in yogurt samples, previous studies showed that the pathogen population decreased faster at abusive temperatures than at cold storage (Belessi et al., 2008; Szczawiński et al., 2016). Similar results were obtained in the current study, where *Listeria* population declined in a shorter time at 12 rather than at 4°C. However, in all of the studies mentioned earlier, the survival of *Lb. monocytogenes* was monitored only after using the typical yogurt culture, in contrast to the current study, where survival of *Listeria* was monitored using the typical culture with the addition of a *Lactobacillus* strain with probiotic potential.

*Lb. monocytogenes* strains are widespread in the environment, and previous studies have revealed that in food processing plants, a high diversity of strains coexists (Almeida et al., 2013; Barancelli et al., 2014). Because foods may be contaminated with more than one strain of the pathogen, it is of great importance to investigate strain competition and selection in different conditions (i.e., storage temperature, packaging conditions, microbiota, etc.), a goal achieved in the present study. It was underlined that the different survival rates of each strain depended on storage time and temperature, as well as on the presence or absence of the probiotic *Lactobacillus* strain. Previous studies dealing with the inter-strain competition of *Lb. monocytogenes* discovered that the occurrence of different strains in the same food environment might trigger strain competition. The strains can be categorized as weak or/and strong competitors, whose growth can or cannot be influenced by the presence of other strains and can contribute to uneven growth of strains in the food (Zilelidou et al., 2015, 2016). Lastly, the observations of Borucki et al. (2003) after screening 80 strains of *Lb. monocytogenes* highlighted the differences between phylogenetic divisions of the pathogen; strains belonging to serotypes 1/2a and 1/2c formed more biofilm than strains belonging to serotypes 1/2b and 4b.

In total, the findings of the current study showed that the addition of the probiotic culture in the artificially contaminated samples resulted in a faster reduction of the pathogen population during storage than in control samples. However, *Listeria* pre- and post-processing contamination, as well as the inter-strain variation, which can include more persistence strains in the food environment, can be a challenge for the dairy industry to confront. Strict sanitary conditions during processing, hygiene of personnel, control of persistence strains, and preventing cross-contamination are critical key factors assuring the microbial safety of the products during manufacture and retail storage.

### Physicochemical Analysis

**FIGURE 4:**
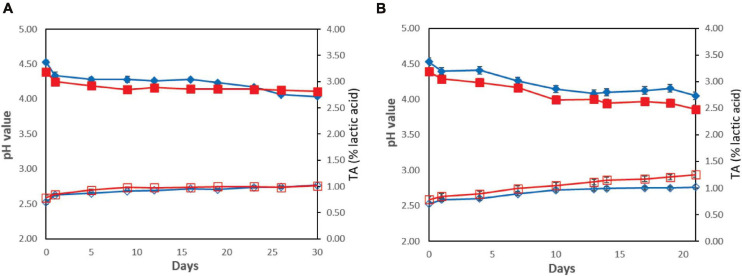
Changes in yogurt samples during storage without (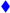) or with (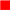) addition of *Lb. plantarum* T571 in pH values (primary axis) and without (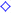) or with (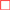) addition of *Lb. plantarum* T571 in titratable acidity (secondary axis) (% lactic acid) at 4°C **(A)** and 12°C **(B)**.

The results for pH and TA measurements during yogurt storage 4 and 12°C are presented in Figure 4. The pH values after the end of the fermentation process were 4.39 ± 0.04 for the probiotic case and 4.53 ± 0.03 for the control case. During storage, the pH values decreased and, by the end of storage, were found to be 4.11 ± 0.01 and 4.04 ± 0.01 for the probiotic and control cases, respectively, at 4°C (*P* > 0.05), whereas at 12°C, lower pH values (*P* < 0.05) were recorded (3.86 ± 0.02 and 4.05 ± 0.01 for the probiotic and control cases, respectively; Figure 4). TA values were found to be 0.79 ± 0.05% of lactic acid for the probiotic case and 0.70 ± 0.04% for the control case after the fermentation. During storage at 4°C, TA values were lower in comparison with the higher temperature for both the probiotic and control cases (*P* < 0.05). In contrast, during storage at 12°C, TA was found slightly higher in yogurt inoculated with the probiotic strain, in comparison with that in control samples (*P* < 0.05). By the end of storage, TA was approximately 1.02% at control samples, whereas the corresponding values of TA were approximately 1.25% for probiotic samples.

In brief, the pH values decreased during the storage of yogurts due to post-acidification, whereas TA increased, a phenomenon that was more intense at the higher storage temperature (12°C). The drop of the pH during the shelf life of yogurts can be attributed to the residual activity of the microorganisms, a result that was also recorded in previous studies. In this context, Li et al. (2017) observed a drop in the pH values during the first 14 days of storage, whereas from the 14th and until the 21st day of storage, pH remained stable. In addition, lower pH values and higher TA were obtained for the probiotic yogurts compared with those in the control. Saxami et al. (2016) also observed lower pH values in the probiotic yogurts compared with those in the control ones. Similar results were noted by Mani-López et al. (2014), who observed pH values of 4.3 to 4.0 after 35 days of cold storage in various combinations of probiotic yogurts when the initial pH was close to 4.6. Moreover, Zhou et al. (2019) noted enhanced post-acidification during cold storage of probiotic yogurt compared with the control. Subsequently, in the studies mentioned earlier, the viability of the probiotic microbiota was maintained to counts over 6 log CFU/g, indicating that low pH did not affect the cell viability, a result that was evident in the present study too.

### Sensory Evaluation

The sensory evaluation of yogurt samples during storage at 4 and 12°C was performed at the same time intervals as for microbiological analysis. The results showed that the end of yogurts’ shelf life depended on the case (control or probiotic) and on the different storage temperatures and scores that generally increased over the storage period (data not shown). Concerning the cold storage, the samples were categorized as “marginal” after 16 days of storage for both the probiotic and control cases, and the shelf life of yogurts was determined on the 26th and 23rd days for the probiotic and control samples, respectively. In addition, the panelist’s mentioned that by the time of sensory rejection, the samples had a sour taste and acidic odor. No other organoleptic attribute (appearance and texture) was affected by the time of sensory rejection. Furthermore, at the end of shelf life, the values of pH and TA were found close to 4 and 1% of lactic acid, respectively, in both cases. At 12°C, the samples were characterized as “marginal” after 7 days of storage in both cases, whereas the shelf life was 13 and 14 days for the probiotic and control samples, respectively.

An important aspect of the probiotic cultures used to manufacture food products is to not adversely affect product quality and to not alter its sensory properties (Li et al., 2017). However, it is expected that the added probiotic culture may develop different patterns of flavor and texture; consequently, it is of importance to determine the sensory changes occurring in the product, specifically when a mixture of LAB is being used (Mani-López et al., 2014). In the current study, the probiotic strain did not alter the texture or the appearance of the products; however, it contributed to a mild acidic taste, after few days of storage, without affecting the total taste scores. Zhou et al. (2019) recorded lower scores for the probiotic yogurt with *Lb. helveticus* H9 in flavor and texture, but the overall changes in the sensory quality were not significantly affected compared with that in the control samples. On the other hand, Mani-López et al. (2014) reported that consumers did not identify texture or flavor differences between the control and the probiotic yogurts, and also, no significant differences in the preferences among the yogurts were recorded.

### Fourier Transform Infrared Analysis

**FIGURE 5:**
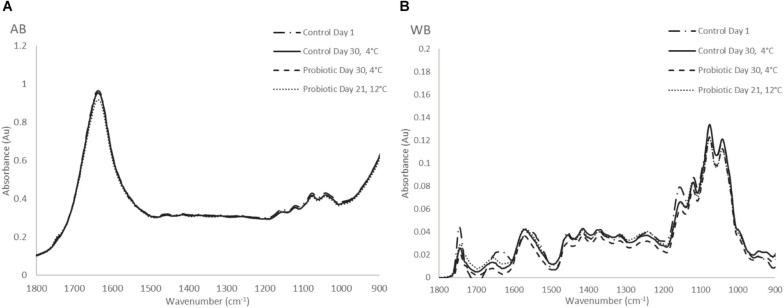
**(A)** Raw FTIR spectra of air background (AB), and **(B)** water background (WB) collected from yogurt samples during storage at 4 and 12°C.

Typical FTIR spectral data (AB and WB) in the range of 1,800–900 cm^–1^ collected from probiotic and control yogurt samples stored at 4 and at 12°C are shown in Figures 5A,B, respectively. Visual observation of Figure 5A did not provide feature peaks that could reflect the difference in quality at the beginning and at the end of shelf life of yogurt samples (AB) at both temperatures, as the spectra were observed to be similar. A major peak was observed at 1,640 cm^–1^ reflecting water (O–H) and amide I band (80% C = O stretch, 10% C-N stretch, and 10% N–H bend). Minor peaks were observed at 1,150 cm^–1^ (fat C–O stretch, esters C–O–C, lactose carbohydrates C–O stretch, and –NH_2_ deformation), 1,110 cm^–1^ (riboses C–O stretch, amines NH_2_ rock/twist), 1,065 cm^–1^ (nucleic acids and phospholipids PO_2_ symmetric stretch/C–O stretch), and 1,040 cm^–1^ (lactose, carbohydrates C–O stretch) (Chen et al., 1998; Wu et al., 2006; Böcker et al., 2007; Pappas et al., 2008; Nicolaou et al., 2010; Aernouts et al., 2011).

On the other hand, at the WB spectra, the major peaks that were observed were found at 1,157 cm^–1^ (fat C–O stretch, esters C–O–C, carbohydrates C–O stretch, and −NH_2_ deformation), 1,111 cm^–1^ (riboses C-O stretch, amines NH_2_ rock/twist), 1,065 cm^–1^ (nucleic acids and phospholipids PO_2_ symmetric stretch/C-O stretch), 1,030 cm^–1^ (amines, free amino acids, and C–N stretch). Minor peaks of the spectra were observed at 1,750 cm^–1^ (C = O stretch of esters and organic acids), 1,640 cm^–1^ (amide I band), 1,550 cm^–1^ (amide II band, 40%CN stretch, 60%NH bend vibration), CH_2_ bending (1,456 cm^–1^) and 995 cm^–1^ (a, b, pyranose compounds- ring vibration, aromatic carboxylic acids) (Ramsaran et al., 1998; Böcker et al., 2007; Pappas et al., 2008). The same trend was observed for samples stored at 12°C, where absorbance (AU) was higher compared with cold storage. In total, no obvious feature peaks were observed to reflect the difference in the quality between yogurt samples; therefore, to quantify the biochemical changes occurring in the samples, data analytics were introduced.

PLS analysis was applied for the prediction of the microbial counts and the sensory scores using only the selected wavenumbers through peak selection (b coefficients). Therefore, data evaluation focused on specific regions of the spectrum. In greater detail, the selected region of the spectrum using microbiological data for prediction was found to be different compared with the corresponding region for the sensorial data (Supplementary Figure 1). Regions at 1,745 cm^–1^, 1,240–1,300 cm^–1^ (corresponding to amide III, C–N stretch, N-H bend, and C = O–N bend), 1,200–1,240 cm^–1^ [corresponding to lipids, nucleic acids (asymmetric PO_2_– stretch), amide III, and amines from free amino acids (C–N stretch)], 1,140–1,170 cm^–1^, 1,079–1,117 cm^–1^, and 980–1,037 cm^–1^ [corresponding to polysaccharides (C–O stretch), and amines (CN stretch)] were considered as the most important wavenumbers (Böcker et al., 2007; Pappas et al., 2008) (Supplementary Figure 1). Former studies stated that polysaccharides dominate in the region between 1,200 and 900 cm^–1^ (C–O stretch), and among them, lactose and glucose display characteristic peaks at 1,159 and 1,035 cm^–1^, respectively (Moros et al., 2006; Păucean et al., 2014). However, Păucean et al. (2014) mentioned that glucose was found only in unfermented milk, whereas phosphates decreased during fermentation due to microbial activity. In addition, esters, lactic acid, and fatty acids concentration increased after milk fermentation, as it was illustrated in the FTIR spectra of the aforenoted study (Păucean et al., 2014).

Good correlations were found after validation of the PLS-R (prediction) models between FTIR estimates (AB and WB) and microbiological (TVC, LAB, and lactic cocci/*S. thermophilus*) (Table 1) and sensory analysis data (actual values). Contrarily, the performance of *Lb. monocytogenes* PLS-R prediction model was found unsatisfactory (Table 1). Regarding the model indices for the microbial count’s estimation (excluding *Lb. monocytogenes*), it was found that the B*f* values were generally very close to unity, indicating good agreement between observations and predictions, i.e., no systematic over- or underprediction of the microbial counts. Moreover, based on the A*f* values, the average deviation between predictions and observations of the various microbial groups enumerated were 2% for lactic cocci/*S. thermophilus*, LAB, and TVC (Table 1). In addition, the accuracy of prediction of the model’s validation was found to be 100% for all the microbial groups except the pathogen, where it was estimated at 34 and 30% for AB and WB spectra, respectively. However, yogurt is a fermented product, and the counts of the examined microbiota did not present a high range (±1 log CFU/g) throughout shelf life, as TVC, LAB, and lactic cocci/*S. thermophilus* were maintained close to 7.5 log CFU/g until spoilage. In addition, SVM-R models were built using normalized data in an attempt to correlate spectral data (AB and WB) with microbial counts and sensory scores. The performance indices of the SVM-R models for the externally validated dataset are presented in Table 1. The average calculated values of the B*f* for the different microbial groups were all close to 1, whereas the A*f* value was estimated at 2% for lactic cocci/*S. thermophilu*s, LAB, and TVC, wherein for *Lb. monocytogenes*, the corresponded value was estimated at 49%, resulting in unsatisfactory predictions (Table 1). Regarding R^2^ values, the variation between the different estimated microbial groups for AB and WB ranged between 0.30 and 0.72 for the PLS-R models and 0.41 and 0.68 for the SVM-R models, whereas the corresponding RMSE values were 0.16 and 1.92 and 0.17 and 1.76 for the PLS-R and SVM-R models, respectively.

**TABLE 1:** Comparison of calculated performance indices for prediction of microbial groups in yogurt samples using predicted estimates of external validation dataset from partial least squares—regression (PLS-R) and support vector machines—regression (SVM-R) models based on FTIR spectral data [air background (AB) and water background (WB)].

		PLS-R					SVM-R				
Spectra	Microbial group	B*f*	A*f*	RMSE	R^2^	% Accuracy of prediction	B*f*	A*f*	RMSE	R^2^	% Accuracy of prediction
AB	TVC	1.00	1.02	0.16	0.72	100.00	1.00	1.02	0.17	0.68	100.00
	LAB	1.00	1.02	0.21	0.52	100.00	1.00	1.02	0.24	0.45	100.00
	lactic cocci/streptococci	1.01	1.02	0.22	0.38	100.00	1.00	1.02	0.21	0.43	100.00
	*Listeria monocytogenes*	1.28	1.70	1.86	0.34	30.41	1.08	1.49	1.63	0.47	55.41
WB	TVC	1.00	1.02	0.20	0.54	100.00	1.00	1.02	0.18	0.63	100.00
	LAB	1.00	1.02	0.21	0.60	100.00	1.00	1.02	0.24	0.48	100.00
	lactic cocci/streptococci	1.00	1.02	0.23	0.34	100.00	1.00	1.02	0.20	0.51	100.00
	*Listeria monocytogenes*	1.26	1.70	1.92	0.30	32.43	1.23	1.66	1.76	0.41	33.11

*TVC, total viable counts.*

Regarding the sensory scores, the PLS-R model exhibited good performance, with the coefficients of determination (R^2^) being 0.89 and 0.86 and the RMSEs 0.25 and 0.29, for the AB and WB spectral data, respectively. The SVM-R also displayed good performance, with values of R^2^ ranging from 0.83 to 0.80 and RMSEs 0.24 and 0.34 for the AB and WB spectral data, respectively.

Finally, the SVM classification model’s confusion matrix for the sensory scores is presented in Supplementary Table 1, and results were satisfactory regarding the accuracy of prediction (92.57 and 87.51%) and sensitivity, i.e., 94.23 and 92.31% for fresh, 97.50 and 87.50% for marginal, and 92.86 and 85.71% for unacceptable yogurt samples for AB and WB spectral data, respectively. In addition, the SVM-R model’s confusion matrix for the sensory classes exhibited classification rates of 84.61% for fresh, 92.50 and 95.00% for marginal, and 78.57 and 85.71% for unacceptable yogurt samples for AB and WB spectral data, respectively (Table 2). Similar results were acquired for the PLS-R model, i.e., 90.38 and 84.61% for fresh, 95 and 85% for marginal, and 98.21% for unacceptable yogurt samples for AB and WB spectral data, respectively, and are presented in Table 2.

**TABLE 2:** Confusion matrix of partial least squares—regression (PLS-R) and support vector machines—regression (SVM-R) models regarding sensory quality discrimination of yogurt samples based on FTIR spectral data [air background (AB) and water background (WB)].

From/To	Fresh	Marginal	Unacceptable	Total	Sensitivity (%)
AB					
Fresh	47	5	0	52	90.38
Marginal	0	38	2	40	95.00
Unacceptable	0	1	55	56	98.21
	Correct classification (accuracy, %)				94.59
WB					
Fresh	44	8	0	52	84.61
Marginal	2	34	4	40	85.00
Unacceptable	0	1	55	56	98.21
	Correct classification (accuracy, %)				89.86
AB					
Fresh	44	8	0	52	84.61
Marginal	3	38	0	40	92.50
Unacceptable	0	12	44	56	78.57
	Correct classification (accuracy, %)				85.13
WB					
Fresh	44	8	0	52	84.61
Marginal	1	38	1	40	95.00
Unacceptable	0	8	48	56	85.71
	Correct classification (accuracy, %)				87.84

Very limited research data are available regarding the use of FTIR spectroscopy in yogurt, with most of the published studies being related to the determination of nutritional parameters or/and adulteration issues. The available data, specifically referring to the application of FTIR spectroscopy in the evaluation of yogurt shelf life, are even scarcer. To our knowledge, the only study reporting on the potential of FTIR spectroscopy for the quantitative monitoring of the yogurt production and the possible deviation of quality parameters is the work of Grassi et al. (2013), where Fourier transform near-infrared spectroscopy was used for the monitoring of lactic fermentation and the estimation of conventional quality parameters. Their results showed that Fourier transform near-infrared spectroscopy combined with principal component analysis was able to describe the curd development during the milk fermentation and monitor in-line the texture of the fermented products (Grassi et al., 2013).

The results of the present study contribute to the enrichment of the currently available information by demonstrating that FTIR spectroscopy is a promising method also for the assessment of the microbiological spoilage and the sensory status of yogurt based on the infrared spectra of yogurt.

## Conclusion

According to the recommendations of various regulatory agencies worldwide, minimum consumption of cell counts per serving, i.e., 10^7^–10^9^, is essential to exert probiotic activity to the host. The findings of the present study demonstrated that the probiotic *Lb. plantarum* T571 strain added in yogurt was viable and in adequate population levels for conferring a health benefit to the consumer, throughout the yogurt storage at 4 and 12°C, and with reference to sensory scores, the addition of this strain led to quality products with desirable organoleptic characteristics. With respect to its contribution to the product’s safety, the addition of the probiotic strain resulted in the reduction of the pathogen’s population in a shorter time compared with the control. Additionally, FTIR spectroscopy appeared to be a promising tool for the rapid and noninvasive monitoring of the microbial counts (TVC, LAB, and lactococci/*S. thermophilus*) and the sensory scores throughout the shelf life of yogurt samples.

However, it would be essential for the dairy industry to provide evidence that the applied probiotic strains will be viable and in adequate numbers throughout the shelf life of the new products. In addition, the population level of the probiotic strains in various commercial products indicated as “probiotic” should be validated by the inspection authorities. Finally, future clinical studies are needed to prove that the probiotic cells can survive through the gastrointestinal tract of the host to provide the associated health benefits.

## Data Availability Statement

The raw data supporting the conclusions of this article will be made available by the authors, without undue reservation.

## Author Contributions

AA, OP, and NC: conceptualization/methodology. AA, OP, and VK: investigation. AA and OP: software formal analysis. OP: writing—original draft preparation. AA, NC, and CT: writing—review and editing. NC and CT: project administration/funding acquisition. All authors contributed to the article and approved the submitted version.

## Conflict of Interest

The authors declare that the research was conducted in the absence of any commercial or financial relationships that could be construed as a potential conflict of interest.
