# Supplementary material for: Use of Fourier Transform Infrared Spectroscopy for Monitoring the Shelf Life and Safety of Yogurts Supplemented With a Lactobacillus plantarum Strain With Probiotic Potential
Source: Front Microbiol. 2021 Jun 28;12:678356. doi: 10.3389/fmicb.2021.678356 (PMC8273496; doi:10.3389/fmicb.2021.678356)
Supplement: Supplementary file 1 [file Data_Sheet_1.pdf]

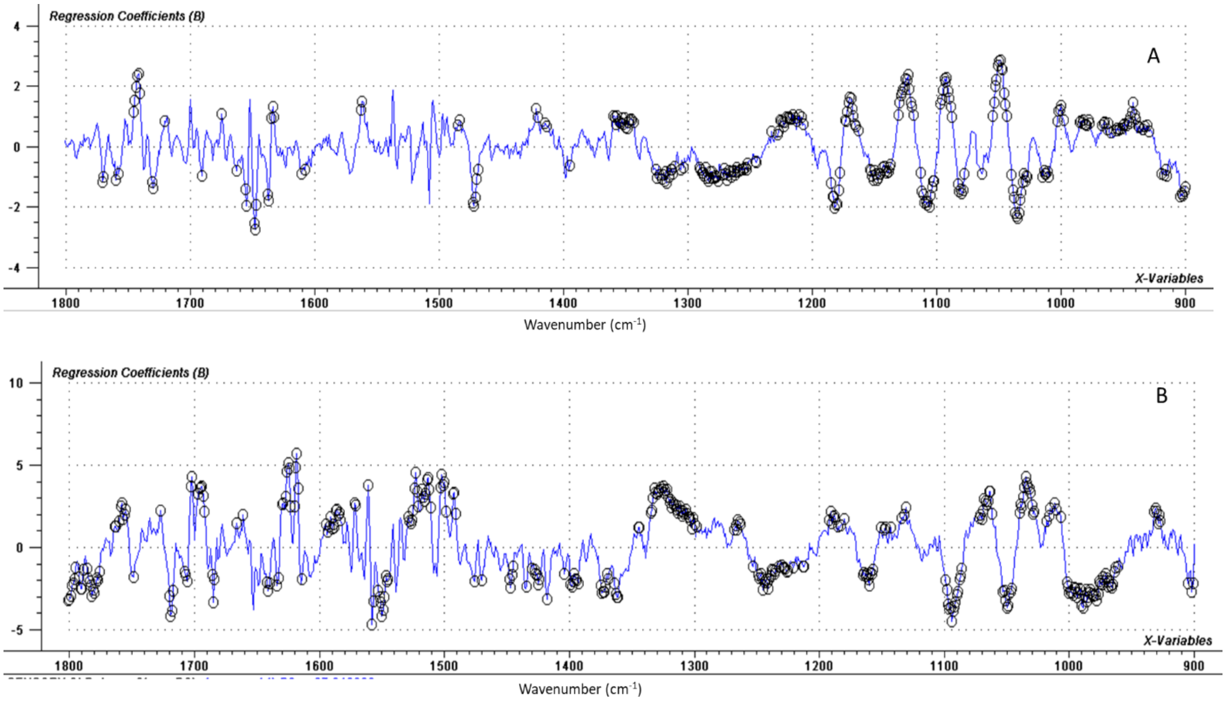

Supplementary Figure 1: Plot of b-coefficients (regression coefficients) over the entire spectral range 1800–900 cm^{-1}. The selected intervals of wavenumbers considered in calculations are marked with open circle symbols. A: Total Viable Counts of Air Background (AB) spectral data B: Sensory data of Air Background (AB) spectral data

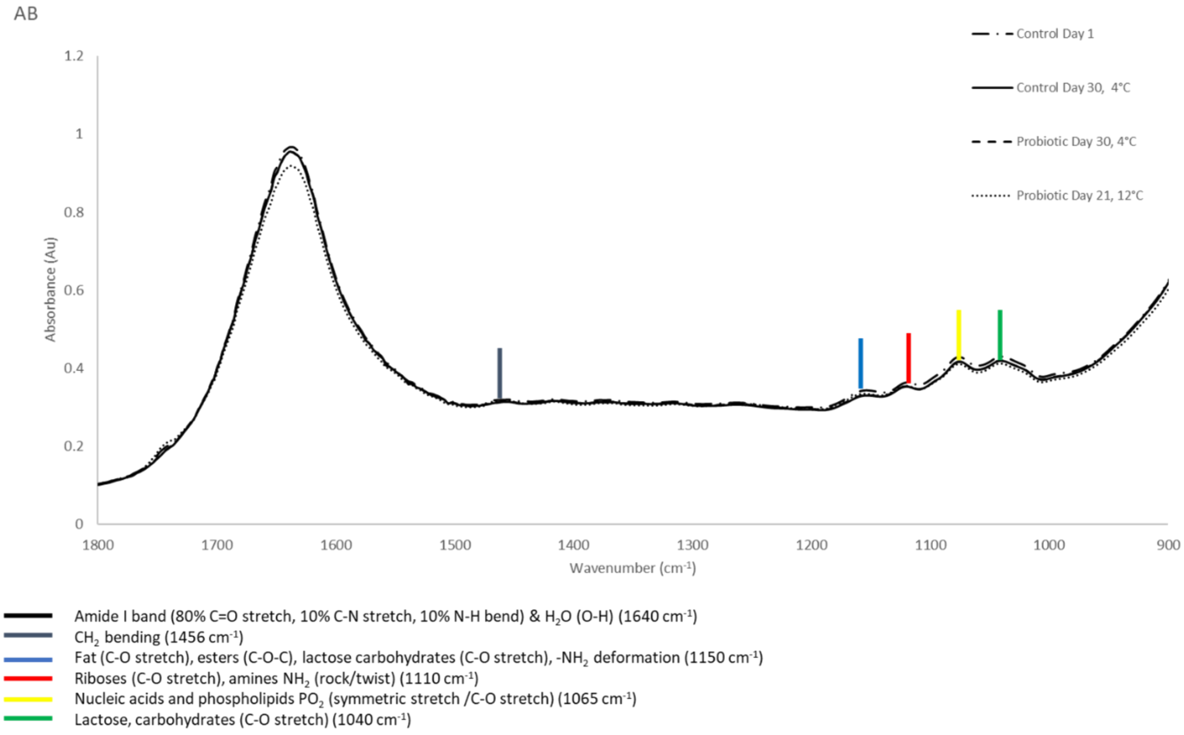

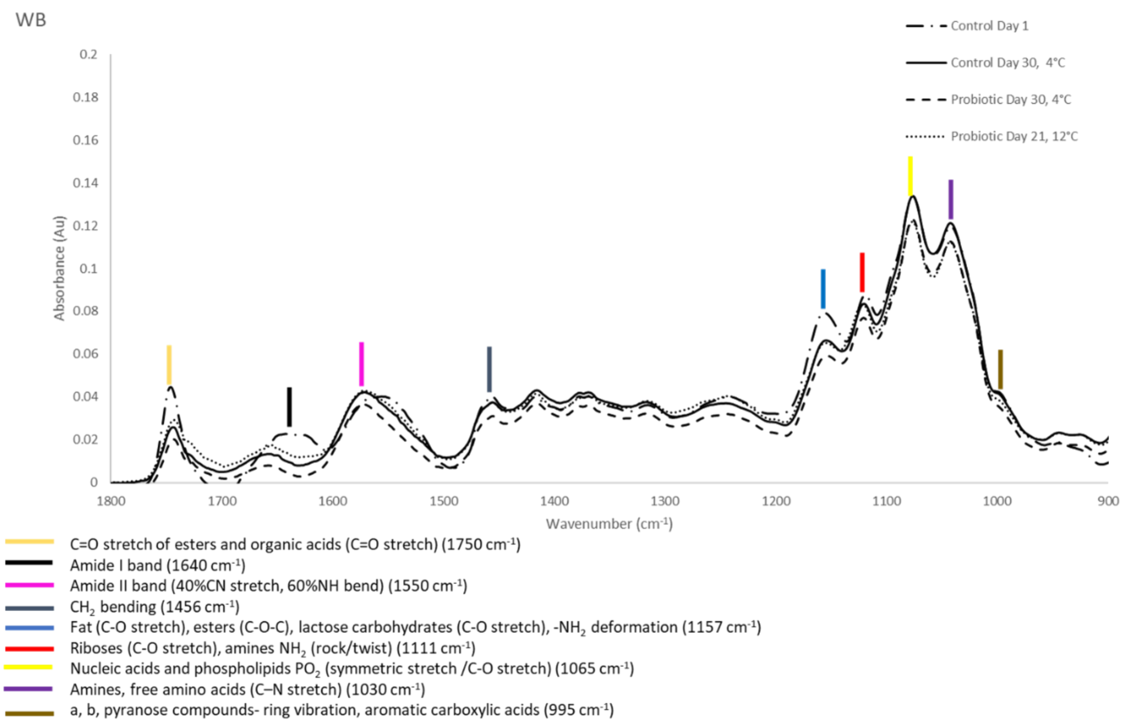

Supplementary Figure 2: Raw FT-IR spectra of Air Background (AB) and Water Background (WB) collected from yogurt samples during storage at 4 and 12 °C. Important functional groups and their region of absorbance are highlighted.

Supplementary Table 1: Confusion matrix of the Support Vector Machines Classification (SVM-C) models regarding sensory quality discrimination of yogurt samples based on FTIR spectral data (Air Background - AB and Water Background - WB).

From/ To	Fresh	Marginal	Unacceptable	Total	Sensitivity (%)
AB					
Fresh	49	1	2	52	94.23
Marginal	0	39	1	40	97.50
Unacceptable	3	1	52	56	92.86
		Correct classification (accuracy, %)			92.57
From/ To	Fresh	Marginal	Unacceptable	Total	Sensitivity (%)
WB					
Fresh	48	3	1	52	92.31

Marginal	1	35	4	40	87.50
Unacceptable	0	8	48	56	85.71
		Correct classification (accuracy, %)			88.51
